# Environmental Injustice and Electronic Waste in Ghana: Challenges and Recommendations

**DOI:** 10.3390/ijerph21010025

**Published:** 2023-12-23

**Authors:** Anuli Njoku, Martin Agbalenyo, Janaya Laude, Taiwo Folake Ajibola, Mavis Asiwome Attah, Samuel Bruce Sarko

**Affiliations:** 1 Department of Public Health, College of Health and Human Services, Southern Connecticut State University, New Haven, CT 06515, USA; laudej2@southernct.edu; 2 Southwestern AHEC, Inc., 5 Research Drive, Shelton, CT 06484, USA; agbalenyom1@southernct.edu; 3 Department of Public Health Sciences, UConn School of Medicine, UConn Health, 263 Farmington Avenue, Farmington, CT 06030, USA; ajibola@uchc.edu; 4 Department of Environmental Health and Sanitation, Akuapem North Municipal Assembly, Akropong-Akuapem P.O. Box 100, Ghana; maattah002@st.ug.edu.gh; 5 Department of International Development, Presbyterian University, Akropong-Akuapem P.O. Box 393, Ghana; sbsarko@presbyuniversity.edu.gh

**Keywords:** electronic waste, e-waste, recycling, environmental health, Agbogbloshie, Ghana, environmental justice, COVID-19, Africa, health disparities, pollution

## Abstract

Electronic waste (e-waste) or discarded electronic devices that are unwanted, not working, or have reached their end of life pose significant threats to human and environmental health. This is a major concern in Africa, where the majority of e-waste is discarded. In the year 2021, an estimated 57.4 million metric tons of e-waste were generated worldwide. Globally, COVID-19 lockdowns have contributed to increased e-waste generation. Although Africa generates the least of this waste, the continent has been the dumping ground for e-waste from the developed world. The flow of hazardous waste from the prosperous ‘Global North’ to the impoverished ‘Global South’ is termed “toxic colonialism”. Agbogbloshie, Ghana, an e-waste hub where about 39% of e-waste was treated, was listed among the top 10 most polluted places in the world. The discard of e-waste in Ghana presents an issue of environmental injustice, defined as the disproportionate exposure of communities of color and low-income communities to pollution, its associated health and environmental effects, and the unequal environmental protection provided through policies. Despite the economic benefits of e-waste, many civilians (low-income earners, settlers, children, and people with minimal education) are exposed to negative health effects due to poverty, lack of education, and weak regulations. We critically examine the existing literature to gather empirical information on e-waste and environmental injustice. Comprehensive policies and regulations are needed to manage e-waste locally and globally.

## 1. Introduction

Electronic waste (e-waste) refers to discarded electronic products that are not working and nearing or at the end of their useful life; it poses significant threats to human and environmental health [1,2,3,4]. According to a 2023 World Health Organization (WHO) report, e-waste is the fastest growing stream of solid waste in the world [5]. As per the United Nations, an estimated 57.4 million metric tons of e-waste were generated worldwide in 2021, up from 53.6 million metric tons in 2019 [5,6]. COVID-19 and its associated global lockdowns contributed to a rise in e-waste circulation [7]. Moreover, there has been an inequitable distribution of e-waste. The influx of hazardous waste from the prosperous ‘Global North’ to the impoverished ‘Global South’ has been called “toxic colonialism” [8,9]. While Africa generates the least of this waste, the continent has been the dumping ground for e-waste from the developed world [10]. E-waste collected for recycling in developed countries is often illegally sorted or disposed of in the developing world, including Ghana [11]. For example, Ghana and Nigeria alone received 77% of the e-waste from England and Wales in 2019 [2]. Moreover, West Africa has emerged at the forefront of receiving illegal shipments of unwanted e-waste, harming vulnerable populations as countries such as Ghana, Nigeria, and Ivory Coast have been the unwilling dumpsite for unlawful e-waste [2]. Other African countries with prolific, local production of e-waste include Egypt and South Africa [6]. There is substantiation that approximately 352,474 metric tons (MT) of e-waste are shipped from European Union (EU) countries to developing countries each year [6]. In 2019, Africa generated 2.9 MT of the world’s 53.6 million metric tons of electronic waste. Despite contributing marginally to the production of e-waste, Ghana takes in millions of tons of e-waste yearly, while an estimated 18,300–60,000 MT of e-waste arrives in Lagos, Nigeria, annually [2].

One of the largest and most known e-waste sites in the world was a recycling site located in Ghana’s capital city of Accra. The site, known as Agbogbloshie, served as an integral recycling site for e-waste, received media scrutiny due to the harmful health and safety conditions of the site, and was ranked among the world’s top ten most toxic threats [12,13]. While the Agbogbloshie scrapyard was demolished by the government in Ghana in July 2021 [14,15], several new informal e-waste recycling sites similar to the abolished Agbogbloshie are appearing throughout Accra and nearby cities [16].

The discard of e-waste in Ghana fuels environmental health disparities and presents an issue of environmental injustice. This is indicated by communities of color and those with low income being exposed to poorer environmental quality and social inequities, experiencing more sickness and disease than wealthier, less polluted communities and being afforded less environmental protection through policies [17]. Moreover, the e-waste situation in Ghana may be challenging to control because third-world countries often do not have the resources to reject importation or manage electronic waste effectively [2].

Furthermore, most e-waste workers have increased exposure to hazardous chemicals due to using crude methods to burn and extract valuable metals from e-waste without using personal protective equipment [1]. Despite the economic benefits of e-waste, many civilians such as low-income earners, settlers, children, and people with minimal education are disproportionately exposed to its health effects due to poverty, lack of education, and lax regulations [1,6,18,19]. Children are especially vulnerable to health risks because they are closer to the ground and ingest a relatively high amount of chemicals in relation to their body size [6].

The aim of the paper is to critically examine the disproportionate human and health impacts of electronic waste in Agbogbloshie, Ghana, and how this occurrence constitutes an environmental injustice. We carefully examine the existing literature to gather empirical information on e-waste and environmental injustice, the human and environmental health effects of e-waste production in Ghana, COVID–19 impacts on e-waste creation, and recommendations for e-waste disposal. Comprehensive policies and regulations are needed to manage e-waste locally and globally. Findings from this study may provide decision makers with information on the state of electronic waste management challenges and the policy recommendations for electronic waste in Ghana.

## 2. Electronic Waste

**Figure 1:**
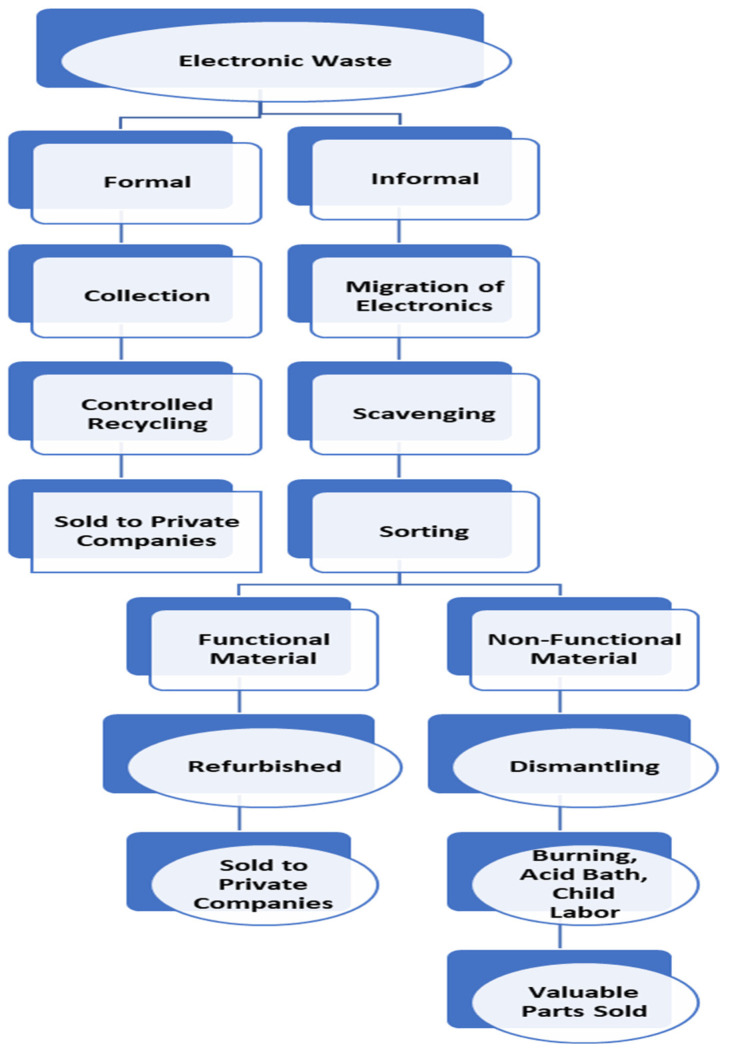
Formal vs. informal E -waste map [3].

Electronic waste can be defined as “broken down devices that are not useful for purposes they were intended for” [1]. It has also been noted by the Basel Action Network that “once discarded, everything with an electrical cord becomes waste” [20]. Sources of electronic waste include computer sets, cathode ray tubes, acid batteries, connectors, flat-screen TVs, chrome plating, printed wiring boards, printer inks and toners, fluorescent lamps, tapes and floppy disks, thermoplastics, and insulators [1]. Discarded electronic and electrical equipment that comprise e-waste include different mixtures of non-hazardous metals (steel and iron), hazardous metals (mercury, lead, arsenic, chromium, cadmium, copper, cobalt, nickel, and lithium), precious metals (silver, gold, platinum, and palladium), and rare-earth metals (indium, tantalum, praseodymium, and neodymium) [21,22]. They also contain chemicals of organic and inorganic nature that can harm the environment and contribute to global pollution through landfill disposal and aerosols released into the atmosphere [21]. Moreover, waste can be divided into informal and formal waste [3] (see Figure 1).

### 2.1. Formal E-Waste Management

Formal electronic waste processes are more expensive, though less physically demanding. Examples of formal electronic waste methods can be examined through waste management companies such as Zeal Environmental Technologies. Located in Takoradi, Ghana, the firm offers services such as the incineration of materials and refrigerator degassing. The company follows both U.S. and Ghana Environmental Protection Agency (EPA) guidelines [23] and is certified by SGS Ghana, a testing, inspection, and certification company, and the United Kingdom Accreditation Service (UKAS). As opposed to directly burning materials, incineration should only burn combustible materials that do not release toxic elements above governing limits [24]. Degassing a refrigerator involves “the removal of oil and gas from the cooling system of a domestic refrigerator without spilling anything into the environment” [25] (p. 5).

### 2.2. Informal E-Waste Management

Within the informal e-waste management sector, toxic chemicals such as lead, mercury, beryllium, and cadmium are released from the dismantling of material, creating an unhealthy environment in which to function [3]. Despite the dangers of informal e-waste processes, the dismantling and refurbishing of waste provides employment opportunities for the residents of Agbogbloshie [26]. In some countries, informal e-waste workers have been found to be less than 16 years old and as young as 8 years old [1]. Informal e-waste management methods include scavenging, sorting, dismantling, and reselling (Figure 1). Waste picking and burning of wires to separate copper and other valuable parts are performed without protective equipment such as gloves, eye-gear, or masks [3]. Scrap dealers build bulks of waste to sell, and refurbishers use old electronic waste to rebuild other electronics [27]. Lastly, between the informal and formal treatment, a public or private formal sector employee collects the waste [3,28]. Moreover, balancing evidence from the North and on-the-ground developing countries’ realities for improved management, informality is an integral part of the economic chain of production, distribution, and employment, with enterprises and workers being at some point on the continuum between pure formal (regulated and protected) and pure informal (unregulated and unprotected), with the ‘formal’ and ‘unregulated’ ends of the continuum being dynamically linked [29].

## 3. History of Agbogbloshie

Agbogbloshie, Ghana, is within a vibrant informal settlement and economy where residential, commercial, and industrial zones overlap, with prevailing land rights struggles dating back to the colonial period [9]. The economic growth of Agbogbloshie is traceable to business in terms of buying and selling food items such as vegetables, root and tuber crops, grains, fish, and meat—onion and yam are the major food items sold there [1]. The Agbogbloshie scrapyard, also known as Old Fadama, was a wetland in the 1960s [9]. Due to a decrease in agricultural opportunity and intertribal conflicts in Ghana’s northern territory, people moved to urban Accra for survival and employment opportunities [9]. The quest of city authorities to decongest the central business district in the early 1990s saw the relocation of hawkers and the Accra yam market to the banks of the Korle lagoon. In 1993, this led to the establishment of the scrap market/scrapyard as various services such as vehicle repair, spare parts trading, welding, auto mechanics, and tire servicing are essential to the operation of vehicles used in transporting food items [9]. By 2001, the land was established as an e-waste site as developed countries used manipulative labeling tactics to gain access to the dumping ground [28].

Described as an area with multicultural inhabitants, workers of Agbogbloshie and others who conduct business there are predominantly people from the northern regions of Ghana [1,9,11,14] and other African countries such as Benin, Burkina Faso, Nigeria, and Togo [1,14]. Agbogbloshie, which is located in the central business district along the banks of the Korle lagoon in Accra, is Ghana’s largest informal e-waste processing and recycling site [26]. It is a built environment with bare surfaces made of concrete and sand, covering an area of 0.313 km^2^ [1]. The recovery and recycling of e-waste was a major activity at Agbogbloshie, and the scrapyard was a key location for e-waste journalism, science, and advocacy, often taking center stage in global e-waste policy work [1,15]. Under the authority of the Greater Accra Scrap Dealer Association, the scrapyard was highly organized with structures, where the processing and recycling of e-waste were carried out by over three hundred small, informal enterprises [14].

In 2014, Agbogbloshie had already received 192,000 tons of waste [28]. The Agbogbloshie scrapyard was demolished by the government in Ghana in July 2021 [14,16], barely a week after a Basel webinar on e-waste management with a focus on Agbogbloshie. The demolition was part of the Greater Accra Regional Minister’s exercise “Let’s Make Accra Work”. This recent development was carried out to pave the way for the president of Ghana’s Agenda 111 hospital initiative, as a section of the site was earmarked for a district hospital [16]. Unfortunately, the demolition of the Agbogbloshie scrapyard was mostly due to a decongestion exercise rather than heavy metal pollution, and the urban poor and the spaces they inhabit have been caught in the crosshairs [16]. The leaders of the scrapyard mobilized to acquire 50 hectares of land at Teacher Mante, 75 km from Agbogbloshie, to serve as the new electronic waste scrapyard [15]. Furthermore, the demolition of the scrapyard did not stop the e-waste work, but instead pushed the practices underground to the inside of homes and closer to where people live. Consequently, instead of one Agbogbloshie plant, many new informal e-waste recycling sites similar to the abolished Agbogbloshie site are emerging throughout Accra and nearby cities [16]. Therefore, the future stability of the e-waste workers and civilians of Agbogbloshie is uncertain.

## 4. Environmental Health Disparities and Environmental Justice

Environmental health disparities exist when communities of color and those with low income are exposed to poorer environmental quality and social inequities and experience more sickness and disease than wealthier, less polluted communities [17]. Environmental racism describes practices, policies, or directives that differentially disadvantage or affect, whether intentionally or unintentionally, individuals, groups, or communities based on their race or color [29]. Environmental equity describes the equal sharing of the burdens and effects of environmental risk and degradation [30]. According to the United States Environmental Protection Agency, “Environmental justice is the fair treatment and meaningful involvement of all people regardless of race, color, national origin, or income, with respect to the development, implementation, and enforcement of environmental laws, regulations, and policies” [31]. Environmental justice also denotes “the equitable treatment and involvement of people of all races, cultures, incomes, and educational levels in the development, implementation, and enforcement of environmental programs, laws, rules, and policies” [32]. It focuses on the disparate impact of hazardous waste and pollutants on racial and ethnic minorities and economically disadvantaged populations and calls for the creation, implementation, and enforcement of environmental regulations, laws, and policies to ensure equal protection from environmental and health hazards, healthy environments, and equal access to the decision making process among all people [31,33]. Furthermore, there is a need to critically examine how policies and systems create and prolong the inequitable exposure to environmental pollutants among communities of color and vulnerable populations, increasing their risk for disease and mortality [34]. The growing creation of e-waste contributes to adverse health outcomes among exposed populations, with the most susceptible being children, pregnant women, and workers in primitive recycling sites [35]. For example, “a child who eats just one chicken egg from Agbogbloshie, a waste site in Ghana, will absorb 220 times the European Food Safety Authority daily limit for intake of chlorinated dioxins”, according to a World Health Organization report [36].

Throughout the world, people that suffer the most from e-waste contamination often contribute the least to the problem [37]. It has been argued that race is a great predominant factor in the location of plants for disposing of toxic waste [38]. While countries worldwide are keenly recognizing the issues around e-waste and establishing legislation, policies, or regulations to govern e-waste, a large portion of e-waste goes unrecorded at its end of life, and a considerable amount of the e-waste is accruing in open dumpsites in several African countries [39]. From a global perspective, Africa generates the least e-waste per capita, behind Oceania, Europe, and America [10]. A large proportion of African countries have the greatest proportion of the people living off of the environment, directly or indirectly, with the e-waste challenge compounded post-COVID-19 [40]. While such threats are well addressed by developed countries with strict law enactments and the establishment of suitable recycling facilities, many developing and underdeveloped nations lack the policies, statutes, technology, and appropriate treatment facilities to manage the e-waste discarded their way [41]. Ultimately, toxic waste dumping in countries of the Global South such as Côte d’Ivoire, Nigeria, and Ghana constitutes environmental racism and requires immediate remediation [40]. For people in Agbogbloshie to reside in an area categorized as toxic and harmful and to face the threat of housing insecurity constitutes an injustice [42]. Uprooting residents without any strategy to increase their wellbeing, allowing them to work in scrapyards without the enforcement of protective equipment, disregarding the health of civilians, and using the informal sector for economic gain are all injustices to the city of Accra. Non-profit organizations have promised to change Agbogbloshie for the better, and yet there has not been steady progress. Examples of campaigns are E-Stewards and Responsible Recyclers by the USEPA. Furthermore, companies use wording and labeling tactics such as “charitable donation” to dump their e-waste while profiting off of the “donation” [42]. This propagates the issue of environmental injustice and calls for prompt resolution.

## 5. COVID-19 Impacts

It is well documented that the COVID-19 pandemic has had a damaging impact on most of the world’s infrastructure, and e-waste disposal systems are not excluded [43]. Since the SARS-CoV-2 virus emerged in December 2019, more than 700 million cases and almost 7 million deaths have been recorded globally [44]. The virus, which spreads via aerosol droplets, through means like coughing and sneezing [45], and fecal–oral and direct contact, led to global lockdowns resulting in supply chain disruptions, exposing the vulnerabilities of health systems previously perceived as stable. Global lockdowns saw the proliferation of work-from-home policies by companies that could afford it, increasing the number of homes with computers and electronic gadgetry, adding to the amount of global e-waste in circulation [7,40]. The disruptions of supply chains from the lockdowns led to a standstill in manufacturing, transportation of essential materials, and plastic, and e-waste recycling. This meant that e-waste recycling previously treated by the formal sector—through scientific methods and while adhering to norms and legislature—would be handled by the informal sector [46]. Most developing companies process e-waste through this method, using unscientific and unregulated methods, which can have dangerous health consequences for the low-income, uneducated population who handle the waste as a means of livelihood [7,46]. In addition, the pandemic and resulting lockdowns led to a near-halt of transportation, facilities, and e-waste sites, severely impacting the formal sector’s collection and transportation of e-waste for recycling [7,47].

As hypothesized by Dutta and colleagues [7], the identical methods of contamination of both e-waste and the coronavirus are likely to have contributed to the broader spread of the virus in larger populations since the viability of the virus on metals and plastics is about five days. These methods of contamination, such as handling or processing e-waste, contaminated computers, or other contaminated devices, are a significant concern that could proliferate the complexities between e-waste disposal and the spread of the virus. E-waste and the coronavirus also have common health hazards like respiratory problems and damage to the nervous system, orthopedic complications, cancer [7], and significant environmental challenges for increased air pollution.

## 6. How E-Waste Pollutes the Environment

### 6.1. Components of E-Waste

E-waste is a complex mix of about one thousand different materials, including plastics, metals, glass, and resins, and glass [48]. Non-hazardous e-waste includes metals such as iron and steel. Hazardous metals include lead and cadmium. Other hazardous metals include mercury, chromium, cadmium, arsenic, nickel, copper, cobalt, and lithium; precious metals such as gold, silver, platinum, and palladium; and rare-earth metals such as neodymium, praseodymium, tantalum, and indium. E-waste also contains organic and inorganic chemicals which pollute the environment upon waste dumping [7]. These toxic materials, including cadmium, lead oxide, and mercury, can enter the environment when they are dumped in unsecured landfills, affecting the quality of living and non-living things found therein [27]. In Ghana, more than a hundred thousand people live daily with fumes, water, soil, animals, and plants contaminated by chemical pollutants.

The crude techniques to electronic waste recycling, for example, acid lixiviation, cause increases in lead, cadmium, copper, and antimony concentrations in soil. This kind of recycling activity represents serious risks for public health. Previous studies on soil samples from Buriram region, a Thai province, and Qingyuan (a city in Guangdong Province, China) indicate larger metal concentrations at active e-waste recycling sites [49].

### 6.2. E-Waste as a Pollutant

According to a study conducted in 2018 by researchers from Japan and Vietnam who used soil samples and floor dust from five households located in an electronic waste processing zone in Hung Yen province, it was estimated that garden soil and floor dust may be the primary source of lead intake by the sampled households. The leakage of hazardous liquid chemicals such as acid contained in the batteries of cars and other motor part fluids end up infiltrating the soil and heavily stain it, giving it a permanently dark color [1]. This form of contamination is a concern because toxic metals can be transferred to the plants, animals, and finally humans. Groundwater, lakes, rivers, streams, and others which serve as sources of drinking water and homes for aquatic animals can be contaminated by chemical pollutants from e-waste via diverse means. Although the contamination of the Ganges River in India was caused by different factors, activity from e-waste recycling sites was identified as the major source of contamination [50].

Ghana faces serious water contamination problems as a result of chemical pollutants from e-waste. The large amount of electronic waste exposed near natural water sources represents a serious public health problem. The constant exposure of e-waste dumpsites to heavy rains causes water contamination by different pollutants that reach Odaw River and the Korle Lagoon, limiting aquatic life diversity [51]. Aquatic plant and animal species of all forms are adversely affected by e-waste activities, while indirectly affecting humans through the intake of fish and seafood. Also, most heavy metals and organic pollutants found in freshwater and saltwater coasts are detrimental to the behavior, development, growth, metabolism, physiology, and reproduction of several aquatic species [11].

### 6.3. Overall Environmental Health Effects of E-Waste

E-waste poses substantial environmental effects which are very detrimental to the environment. In Ghana, a number of studies have shown the negative impact of e-waste activities on the environment [52,53]. According to Islam and colleagues [54], e-waste constitutes 70% of all toxic and hazardous chemicals in the environment. The concentration of antimony, Cd (cadmium), and Pb (lead) in soils in Agbogbloshie and Koforidua has increased by over 100% due to the burning of electronic waste. The incessant and unregulated burning of e-waste in areas like Agbogbloshie has destroyed the ecosystem and the river, leading to the loss of virtually all aquatic life [55].

The improper handling of electronic waste also threatens food security. Concentrates of elements such as Cd (cadmium) (103.66 mg/kg), Sn (tin) (705.32 mg/kg), Ni (nickel) (72.00 mg/kg), Cu (copper) (202.99 mg/kg), and Pb (lead) (184.44 mg/kg) found at e-waste burning sites Korle Lagoon in Accra are above the threshold for arable soil set by the WHO/FAO [52]. Also, a lagoon close to Agbogbloshie where e-waste is disposed of and burnt was found to contain an elevated level of Cu, Pb, Zn, and Cd [55]. Chemicals from e-waste dumpsites can easily seep through the soil to contaminate the groundwater [54]. Heavy metals found in freshwater bodies and the sea adversely affect the physiology, metabolism, reproduction, development, and growth of aquatic species [56]. For instance, the Odaw River flows right alongside the e-waste dumpsite, and the Korle Lagoon is close to the site. These water bodies are some of the most important catchments of the Accra metropolitan area, covering an area of 250 square km [56]. The Odaw River frequently floods when it rains, transferring surface chemical contamination to adjacent lagoons and rivers, thereby increasing their toxicity level, which adversely affects aquatic life [55].

Huang and colleagues’ study [56] further revealed that higher concentrations of copper, cadmium, lead, iron, and chromium were found in water bodies within the e-waste site catchment, and significantly higher concentrations of polychlorinated biphenyls (PCBs) were found downstream of the e-waste dumpsite relative to downstream of the Accra central business district [57]. Studies have also found elevated levels of heavy metals and organic pollutants in marine life, including fish, downstream of and in urban shores of Accra [57,58]. Furthermore, many toxic chemicals and toxic metals such as lead have been detected in soil samples from surrounding areas of the e-waste dumpsite in Accra [55].

Studies have further indicated that various metals and metalloids, including As (arsenic), Cd (cadmium), Co (cobalt), Cr (chromium), Cu (copper), Ni (nickel), Sb (antimony), and Zn (zinc), can enter the environment and pose tremendous risks, such as soil contamination, through the mechanical dismantling, open burning, and leachate of e-waste materials [59,60]. Studies [59,61] have affirmed that soils in e-waste-burning areas contained metals such as As, Cd, Cu, Pb, and Zn in amounts well above international environmental guidelines for soil quality, such as the Dutch target and intervention levels and the Canadian Council of Environment Ministers (CCME) guidelines [62]. Additionally, a recorded account of a concerned resident at Agbogbloshie revealed, ‘‘in most cases, you smell Agbogbloshie before you see it. At a distance, a billowing black smoke can be seen blanketing the grounds from the market’s chaotic entrance, where a myriad of people go about their day-to-day business. The place is filthy, crammed with people and junk computers, most of which are eventually set on fire ’’ [63] (p. 11).

## 7. Health Impacts of E-Waste and Affected Populations

### 7.1. Health Impacts of E-Waste

Prior to its demolition in July 2021, Agbogbloshie had come into the limelight as one of the biggest, busiest, and most polluted places for e-waste processing in the world [62]. Electronic waste across the world is one of the largest sources of heavy metals and organic pollutants in municipal solid waste [64]. Approximately 53.6 million metric tons of e-waste were generated globally in 2019 and an estimated 82.6% is most likely dumped, traded, or recycled informally [65]. African countries including Ghana are leading the use of electrical electronic equipment, with an accompanying high increase in the generation of e-waste [12]. E- waste is known to generate a wide range of toxic chemicals during dismantling, and these chemicals have been linked to a number of adverse health consequences [1] (see Table 1).

In Agbogbloshie and developing countries, e-waste is manually dismantled by workers in the informal sector to extract and sell valuable components like copper and gold by burning plastic and the insulation tubing on metal wires, which releases harmful pollutants into the air, soil, and lungs of residents and workers in the area [66]. Breathing in toxic fumes from e-waste combustion in open areas has been reported to result in various pulmonary problems like respiratory infections, bronchitis, and the growth of lung scar tissue [66].

Hazardous elements such as iron, lead, and antimony were detected in the urine of the workers at the Agbogbloshie e-waste site in Accra. The samples of urine, blood, and maternal milk of people taken at the e-waste site of Agbogbloshie showed that the levels were beyond the standard threshold [2]. Generally, poor countries are seriously affected by e-waste due to a multiplicity of factors as people in these countries are exposed to hazardous substances through food, dust, smoke, and water due to the environmental degradation of the chemicals released into the environment [67]. Workers who engage in the burning of electronic waste have comparatively higher levels of toxic chemicals in their blood and urine [67]. Redeye and back pain are relatively prevalent among the workers at Agbogbloshie, and the high consumption rate of drugs among the workers has been attributed to poor working conditions and chronic pain [19]. It has also been reported that higher levels of perceived stress and perceived noise exposure were associated with an increased number of occupational injuries among electronic waste recycling workers in Ghana [68].

The majority of workers at e-waste collection sites do not use safe methods in processing waste. The opening, burning, and manual handling of the waste exposes workers to hazardous substances [14]. One study indicated that exposure to lead can lead to health problems such as anemia, coma, and even death [6]. The same study further revealed that cadmium negatively affects health outcomes, reduces cognitive function, and causes social and attention problems [6]. It has also been indicated that exposure to e-waste increases the susceptibility of people to cancer, miscarriage, and neurological damage [2].

Although nobody is immune to the effects of electronic waste, some groups of people are more affected and over-represented. Children are especially susceptible to the health risks of e-waste, and the effects of e-waste exposure on children include reduction in intelligent quotient (IQ) and behavioral abnormalities, including childhood temperament abnormalities. Children are disproportionately affected because they are closer to the ground and also ingest relatively high amounts of harmful substances per their body size [6]. Children are also more susceptible because of their physiology, which involves comparatively low rates of toxin elimination and high intakes of food, water, and air; therefore, the health behaviors of children such as hand-to-mouth activities also make them more susceptible [69]. The exposure of males to e-waste has been reported to cause prostate cancer [35], while workers at e-waste sites face health issues such as cuts, body pains, coughs, and catarrh, among other prevalent health concerns [4].

### 7.2. Demographics of E-Waste Workers

**Figure 2:**
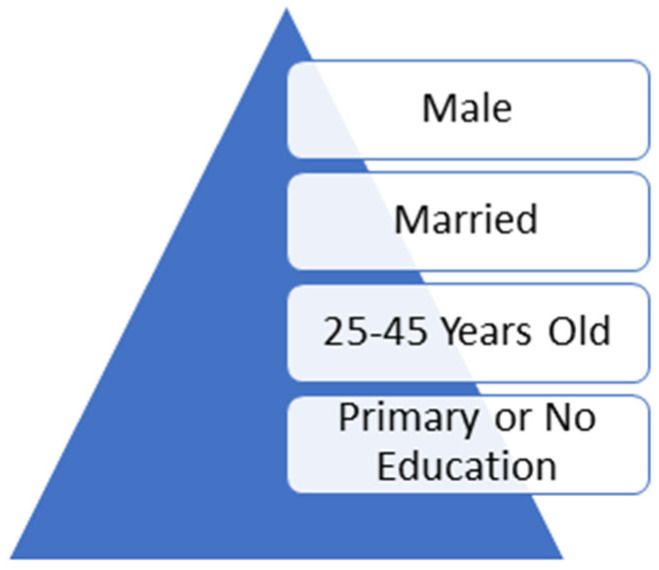
Typical demographics of e-waste workers [18,19].

A study by Adanu and colleagues revealed that most of the e-waste activities at Agbogbloshie have been dominated by people with minimal levels of education (65% of the workers have no formal education) and who are mostly under 45 years of age [1]. Another study [27] further revealed that the average age of the workers on the e-waste site was 21 years. There is also the problem of child labor, as children as young as 5 years are engaged in e-waste activities without recourse to the detrimental health consequences [6]. Overall, studies have corroborated that e-waste workers tend to be male, married, between 25 and 45 years old, and with primary school level or no education [18,19] (see Figure 2).

Most of the scrap dealers at Agbogbloshie tend to be people from northern Ghana and other countries such as Togo, Benin, and Nigeria who are without jobs [1]. These workers spend a lot of their time on the site and hence their exposure to toxins is a huge risk [2]. The relative neglect of northern Ghana has forced a lot of them to move to Agbogbloshie in search of jobs. This situation has been described as slow, and socioecological violence makes them vulnerable [9]. It has been reported that the workers at Agbogbloshie have higher rates of mental problems due to factors such as poverty, which is very prevalent in northern Ghana and other neighboring countries, where the workers come from [19]. However, issues such as stigmatization and violence at Agbogbloshie worsen the psychological and socioemotional condition of the workers [19]. People are exposed to health risks even after working hours. One study revealed that 99.6% of the participants slept close to Agbogbloshie, while 70.7% of the workers slept inside their work shed at the site [67]. On average, workers resided near Agbogbloshie for 6.0 years [67]. Accordingly, the most susceptible group of people includes pregnant women, children, and workers who use crude methods of processing electronic waste [35].

## 8. Enforcement of Regulations

Treaties that regulate the entry of waste into developing countries are the Bamako and Basel Conventions [42]. Founded in 1992, the primary function of the Basel Convention is to prohibit exploitation of developing countries [42]. Similarly, the Bamako Convention was established in 1998 and was meant to protect vulnerable countries, including Ghana, from exploitation. There has been limited enforcement of the regulations [14]. According to Race and Waste: The Politics of Electronic Waste Recycling and Scrap Metal Recovery in Agbogbloshie, Accra, Ghana, the Basel Convention “requires member countries to publicly report details about their exports and imports of hazardous and other waste” [42] (p. 336). Policies elsewhere in the world, for example, Japan, have a “Consumer Pays Model” which requires the consumer of the product to pay recycling fees [70]. Europe has an “Extended Producer Responsibility”, which shifts the responsibility toward the producer of a product and away from municipalities [71].

According to the Handbook of Electronic Waste Management, the Extended Producer Responsibility principles are protection strategies designed to lessen the total environmental impact from a product and its packaging [72]. Extended Producer Responsibility (EPR) principles require the producer to be held accountable for the entire lifespan of a product and to be cognizant of the design and marketing [72] of the product so as to prevent electronic waste. Guidelines of EPR include the formation of collection centers in collaboration with original equipment manufacturers.

In addition, formal strategies under EPR include taking inventory of e-waste stock nationwide through third-party and public–private partnerships [72]. According to the Plastic Revolution Foundation, “Ghana can currently be said to be in the early stages of implementing EPR, with preliminary dialogues being held, an overarching government strategy involving EPR having been launched and approved, and initial producer organization having emerged over the past years” [73].

Overall, while e-waste regulation is more developed in certain parts of the world, only 13 African countries had national regulation to govern the disposal of e-waste in 2019 [65]. An inventory of e-waste legislation within Africa shows that there is still improvement needed in this area [6] (see Table 2).

### Policy Approaches to Effective E-Waste Management

Undoubtedly, electronic waste processing is an emerging lucrative business in the developing world. However, its health impacts cannot be overlooked [74]. In pursuit of livelihood, vulnerable workers sacrifice their health on the altar of economic gains. Although the government of Ghana made efforts to demolish the e-waste site at Agbogbloshie, unsafe e-waste management practices are still thriving in Ghana since their livelihoods depend on this business. It is therefore expedient for the government to provide alternative sources of livelihood for the people. Providing safe economic activities for these people will not only enhance their wellbeing but also protect the physical environment from contamination.

According to Merem et al. [2], developing countries lack the resources to resist the attempts made by the developed world to make their region a dumping ground for e-waste. It will, therefore, be difficult for Ghana alone to fight this battle of toxic colonialism against giant economies such as the United States and Britain. Ghana needs to collaborate with other developing countries in Africa in order to come out with a comprehensive plan on electronic management. Lessons from the Basel Convention and the Bamako Convention teach that being signatories to international conventions is not enough [42]. Developing countries need to pull their resources together to develop and implement policies that will protect them from toxic colonialism. Collaboration among African countries will make them a stronger force that will effectively control the flow of electronic waste entering their region.

As mentioned earlier, countries such as Japan implemented a policy known as the Consumer Pays Model [70]. An e-waste levy can be imposed as a consumer tax on used electronic gadgets. Ghana should implement this policy since it will generate extra revenue, which can be used to manage electronic waste.

One of the means of managing electronic waste is the implementation of Extended Producer Responsibility, which requires producers of electronic waste to bear the cost of waste management [71]. Policymakers should implement this measure as a way of holding the manufacturers accountable. In the case of Ghana, where a huge volume of imported electronic products are obsolete, importers should bear this extended responsibility. The Ghana Revenue Authority and Custom Excise and Preventive Service should implement this levy by imposing it as extra import duties on used electronic goods before the goods are cleared from the ports in Ghana.

According to Oteng-Obabio [70], the Government Act (1994), Act 462, and the Environmental Sanitation Policy of Ghana (1999), which aimed to regulate toxic and radioactive waste, were implemented before toxic colonialism became a major threat to Ghana. In order to provide a tailor-made solution to this emerging problem, the Hazardous and E-waste Control and Management Act 917 was passed in 2016 [14]. However, the awareness level among stakeholders was low due to low level of education and lack of publicity. Our findings suggest that most workers involved in e-waste activities do not have a formal education [1]. A low level of education will consequently affect their subjective judgment and evaluation of the risks of electronic waste. Kiddee, Naidu, and Wrong [75] discussed how policies can only achieve the desired results if the people accept and comply with them. The Environmental Protection Agency must, therefore, involve the people in the implementation of the policies through publicity, education, and community forums. The involvement of the people will promote the acceptance and sustainability of the legislation.

Owusu-Twum [14] contends that economic incentives can be employed to improve the management of electronic waste. In order to prevent the indiscriminate disposal of electronic waste, authorities should provide economic incentives such as cash or discounts for returning used electronic gadgets. The government should establish an official body for this exercise. This will enhance sustainable e-waste practices since the collection and processing of these gadgets are regulated by the government.

Furthermore, our findings also revealed that the e-waste management sector has been dominated by informal workers who mostly use unsustainable management practices [3]. Instead of demonizing informal e-waste practices, regulatory bodies should issue environmental permits to all e-waste workers. These mandatory permits will be issued and renewed based on meeting the standards of EPA [31]. This will protect the environment and the health of the workers. Regulatory bodies should revoke such permits for non-compliance.

## 9. Recommendations for E-Waste Disposal

As discussed in this article, unregulated and indiscriminate e-waste disposal in developing countries like Ghana and Nigeria poses a significant public health risk. Reducing unregulated e-waste disposal and resulting health inequities must be addressed through multifaceted strategy and collaboration between public, business, and government stakeholders. We propose the following recommendations:

1. Promote Awareness and Education: Public health education and promotion efforts should be designed to thoroughly educate informal workers and the general public about the dangers that e-waste poses to the environment and public health and the value of disposing of it properly. Increased knowledge of the dangers of e-waste can be achieved through public awareness campaigns, community education initiatives, and academic initiatives. Since it has been proven that education does not necessarily affect behavioral change, additional interventions that address the financial motivations for informal e-waste handling must be concurrently implemented [66].
2. Enhance Regulations: Governments must create and implement rules that forbid the disposal of e-waste in landfills and waterways. The passing of legislation like the Hazardous and Electronic Waste Control and Management Act, passed by the Ghanaian parliament, must be further enforced to restrict the amount of hazardous and electronic waste imported into developing countries while complying with international accords like the Basel and Bamako Conventions [66].
3. Implement a Formal Recycling Program: Governments should create a formal method for collecting, transporting, and recycling electronic trash. Businesses that take part in the program may receive incentives from the government. Additionally, this will help the local economy and create jobs.
4. Promote Local Manufacturing: To lessen the importation of e-waste, governments should promote the manufacturing of electronic products in Africa. Additionally, this will help the local economy and create jobs.
5. Offer Medical Services: There should be provision of medical assistance to those who have been exposed to e-waste. In regions where e-waste exposure is prevalent, the government can set up clinics for healthcare.
6. Endorse Sustainability: There should be societal efforts to encourage environmentally friendly behaviors, including adopting energy-saving gadgets, prolonging the life of electronics, and using circular economy ideas. This will increase resource efficiency and cut down on the production of e-waste.
7. Encourage Multisectoral Cooperation: To effectively tackle the e-waste crisis, there should be national efforts to encourage cooperation between governments, the commercial sector, civic society, and international organizations. This can involve exchanging financing, technological knowledge, and best practices.

By implementing these proposals, Ghana and Africa may promote sustainable development while reducing e-waste disposal and health inequities. To effectively manage this complicated issue, all parties must be dedicated, collaborative, and persistent. 

## 10. Conclusions

In conclusion, electronic waste (e-waste) poses significant threats to human and environmental health. It is a major concern in the continent of Africa and in countries like Ghana, where a majority of e-waste is discarded. COVID-19 and its related global lockdowns added to a rise in e-waste circulation [7]. Moreover, there has been an inequitable disposal of e-waste, which presents an issue of environmental injustice. Africa has been the dumping ground for e-waste from the developed world, and the influx of hazardous waste from the prosperous ‘Global North’ to the impoverished ‘Global South’ has been termed “toxic colonialism” [1,2]. While an e-waste scrapyard in Agbogbloshie, Ghana, was demolished by the government in 2021, several new informal e-waste recycling sites similar to the abolished site are appearing throughout Accra and nearby cities [16]. Reducing unregulated e-waste waste disposal and addressing resulting health inequities is needed through a multifaceted strategy and collaboration between public, business, and government stakeholders. Recommendations include to promote awareness and education on the dangers of e-waste, enhance regulations to restrict the flow of toxic waste into developing countries, implement a formal recycling program, promote local manufacturing, offer medical services to exposed populations, endorse sustainability, and encourage multisectoral cooperation. A collaborative effort is needed by a multitude of stakeholders to tackle this pervasive issue.

## Figures and Tables

**Table 1:** Chemicals in, main sources of, and effects of human exposure to e-waste [1].

Chemicals	Main Source	Effects on Human Health
Lead (Pb)	Cathode ray tubes (CRT), acid batteries, computer monitors, polyvinyl chloride (PVC) cable.	Brain damage; reduction in fertility in men. Damage to the nervous system, miscarriages, anemia, and kidney problems.
Barium (Ba)	CRT, vacuum tubes.	Paralysis and death.
Beryllium (Be)	Connectors, motherboards and finger clips.	Chronic beryllium disease (CBD). Damages organs such as the heart, lymph nodes, kidneys, skin, spleen, liver, etc.
Polychlorinated biphenyls (PCBs)	Electrical transformers, capacitors of TV sets, computer monitors, radios, and PVC.	Neurobehavior and reproductive problems among infants, adverse effects on reproductive functions, burning sensation, chloracne and related dermal lesions, pigmentation disturbances of skin and nails, erythema and thickening of the skin, and burning sensations.
Cadmium (Cd)	Switches, solder joints, housing coatings, cathode ray tubes, rechargeable batteries.	Lung damage, kidney damage, stomach problems, diarrhea, damage to the central nervous system, and weakened immune system.
Mercury (Ag)	Batteries, flat-screen TV sets, switches, relays, computer housing.	Negative impacts on thinking, memory, attention, language, fine motor skills, and visual–spatial abilities.
Chromium (Cr)	Steel housing of CPU and chrome plating.	Effects include skin rashes, stomach problems and ulcers, respiratory problems, weakened immune systems, kidney and liver problems, negative effects on genetic material, lung cancer, and death.
Nonylphenol (NP)	Insulators, housing, and casing.	Skin and eye problems.

**Table 2:** African countries with e-waste legislation/policy laws [6].

North Africa	Legislation	South Africa	Legislation	Central Africa	Legislation	East Africa	Legislation	West Africa	Legislation
Algeria	No	Angola	No	Cameroon	Yes	Burundi	No	Benin	No
Egypt	Yes	Botswana	No	Chad	No	Comoros	No	Burkina Faso	No
Libya	No	Lesotho	No	Central African Republic	No	Djibouti	No	Cabo Verde	No
Mauritania	No	Madagascar	Yes	Congo	No	Ethiopia	No	Cote d’Ivoire	Yes
Morocco	No	Malawi	No	DR Congo	*	Kenya	Yes	Gambia	No
Tunisia	No	Mauritius	No	Equatorial Guinea	*	Rwanda	Yes	Ghana	Yes
		Mozambique	No	Gabon	No	Seychelles	No	Guinea	No
		Namibia	No			Somalia	*	Guinea Bissau	No
		São Tomé and Príncipe	Yes			South Sudan	*	Liberia	*
		South Africa	Yes			Sudan	No	Mali	No
		Swaziland	No			Tanzania	Yes	Niger	No
		Zambia	Yes			Uganda	Yes	Nigeria	Yes
		Zimbabwe	No					Senegal	No
								Sierra Leone	No
								Togo	No

* No information available on e-waste policy regulation/legislation according to Forti et al. [65].

## Data Availability

No new data were created or analyzed in this study. Data sharing does not apply to this article.
